# The relationship between dietary acid load and intensity of musculoskeletal pain condition: A population‐based study

**DOI:** 10.1002/fsn3.2859

**Published:** 2022-04-05

**Authors:** Niki Bahrampour, Cain C. T. Clark

**Affiliations:** ^1^ Department of Nutrition, Science and Research Branch Islamic Azad University (SRBIAU) Tehran Iran; ^2^ 2706 Centre for Sport, Exercise, and Life Sciences Coventry University Coventry UK

**Keywords:** dietary acid load, muscle pain, net endogenous acid production, pain intensity, potential renal acid load

## Abstract

Pain is a globally prevalent problem, and a comprehension of its pathophysiology is important with respect to patient's health. Musculoskeletal pain conditions (MPs) may be associated with physical, lifestyle, and nutrition status, while dietary acid load (DAL) may be inversely associated with musculoskeletal health in adults. This cross‐sectional study consisted of 175 adults experiencing pain. Anthropometric measurements, physical activity (PA), and pain intensity were assessed via specific questionnaires. Dietary data were collected using a 7‐day 24‐h recall. Foods and beverages were analyzed with Nutritionist IV software for extracting the total energy and nutrients. Net endogenous acid production (NEAP) and potential renal acid load (PRAL) were evaluated for assessing the DAL. Linear regression and Spearman correlation were used to investigate the association of exposure and input variables. Linear regression showed a positive relationship between PRAL and NEAP and pain intensity in the crude model. This significant positive relationship remained after adjusting for all confounders. A lower consumption of potassium, magnesium, vitamin B9 and C, and fiber was seen in the following quartiles of PRAL and NEAP. In addition, MPs intensity and PRAL and NEAP had a weak, positive correlation. This study suggests that a higher DAL may be associated with MPs. However, further research is needed.

## Graphical abstract

This study demonstrates a significant, positive, relationship between higher dietary acid load (DAL) and pain intensity among adults with musculoskeletal pain.

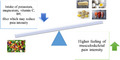

## 1 INTRODUCTION

Pain is a globally prevalent problem, and discerning the pathophysiology of musculoskeletal pain conditions (MPs) is important to support patient's health (Chen & Sehdev, 2019). Pain may persist for more than 3–6 months and is often attributed to nerve damage (Martin & Reid, 2017). The global prevalence of chronic pain is 20.4%, which is higher in women than men (21.7%) and those aged 65 and over (30.8%) (El‐Metwally et al., 2019). Patients can also experience an intense range of pain from mild to severe (Dueñas et al., 2016). Overall, pain can cause, or contribute to, disability, in addition to lifestyle altering changes when treatment becomes ineffective (Institute of Medicine (US) Committee on Pain D and CIB et al., 1987).

Many large studies have suggested that pain intensity, especially MPs, may be associated with physical, lifestyle, and nutrition status (Mills et al., 2019). However, the role of dietary intake in treating and/or prevention of pain intensity is less well known. Choosing a planned individualized diet may help to reduce the complications and improvement of MPs (Elma et al., 2020). Accordingly, Elma et al. found that patients with rheumatoid arthritis pain have a low intake of calcium, folate, zinc, magnesium, and vitamin B6, while pain intensity may be related to fat and sugar intake in these patients (Elma et al., 2020). Dietary acid load (DAL) is defined as the balance of acid/base‐inducing foods (Hayhoe et al., 2020). Problematically, modern Western diets are high in animal products like eggs, meats, cheese, and grains but low in fruits and vegetables (Frassetto et al., 2018), and this popular diet can aggravate chronic pain through pro‐inflammatory cytokine secretion (Dragan et al., 2020).

Meat, eggs, cheese, and cereal grains are acid producing in the body, while base‐inducing foods are fruits and vegetables (Frassetto et al., 2018). For evaluating the DAL, net endogenous acid production (NEAP) and potential renal acid load (PRAL), which is more accurate than NEAP (Mohammadpour et al., 2020), can be used (Cunha et al., 2019). Hayhoe et al. showed an inverse association between PRAL and musculoskeletal health in older adults (Hayhoe et al., 2020), where an acid–base imbalance may be responsible for increasing the inflammation and severity of pain (Zampieri et al., 2014). Moreover, some studies have shown that higher DAL is inversely associated with the health of the muscle mass (Granic et al., 2016). However, no study has evaluated the relationship between the DAL and pain intensity. Thus, the current study aimed to investigate the relationship between DAL and intensity of MPs among Iranian adults.

## 2 MATERIALS AND METHODS

### 2.1 Study population

This is a cross‐sectional study, including 175 men and women. The participants were chosen from among patients expressing pain in physiotherapy and orthopedic clinics, >18 years, in districts 2 and 3 of Tehran, Iran, using multistage cluster random sampling. The sample size was calculated based on the Kelsey formula:

$$N_{\mathrm{Kelsey}}=\frac{{\left(z_{\frac{\alpha }{2}}+z_{\beta }\right)}^{2}p\left(1‐p\right)\left(r+1\right)}{r{\left(p_{0}‐p_{1}\right)}^{2}}$$

Where *α* = 0.05, *β* = 0.2, *r* = 1, with 80% power and 95% confidence interval (CI). The inclusion criteria were having MPs. Exclusion criteria were having a bone fracture in the last 3 months, pregnancy and/or lactation, and psychosomatic disorders. Information on age, gender, education, job, and marital status was collected. In addition, delivery type undergone by of women (cesarean, natural, and no delivery) was assessed via questionnaire. This study was approved by the National Committee for Ethics in Biomedical Research under code IR.IAU.SRB.REC.1399.084. All volunteers were informed about the study and provided written informed consent, prior to participation in the study.

### 2.2 Anthropometric measurement

The weight was measured by digital scales, when participants were fasted for 8 h and were in light clothing, to the nearest 0.1 kg. The height was measured with Seca 216, to the nearest 0.1 cm, with participants in a standing position and unshod. The waist circumference (WC) (cm) and body mass index (BMI) (kg/m^2^) were measured for all participants according to standard techniques.

### 2.3 Pain assessment

The MPs severity was evaluated using the validated McGill Pain Questionnaire, consisting of 20 questions (Khosravi et al., 2013). The intensity of MPs was scored from 0 (no pain) to 78 (severe pain) and was conducted by an expert nurse.

### 2.4 Physical activity assessment

Physical activity (PA) of the participants was evaluated using the short form of the International Physical Activity Questionnaire (IPAQ) (Craig et al., 2003). The metabolic equivalent‐minutes per week (MET‐min/wk) were assessed by summing the activity hours per week. Finally, the variable was divided into three parts: low, moderate, and high activity.

### 2.5 Dietary data collection

Food intakes of subjects were gathered using a 7‐day 24‐h dietary recall through an interview. All foods and beverages consumed were ascertained during the last 7 days. Then, each food and beverage was analyzed for their energy and nutrients with Nutritionist IV (version 7.0; N‐Squared Computing, Salem, OR), a software program modified for Iranian foods (Ghodoosi et al., 2020). The software database was drawn from the United States Department of Agriculture (USDA) food composition tables. In addition, only a total energy range between 800 and 4000 kcal/d was accepted, outside of which, participants were excluded (Banna et al., 2017). PRAL and NEAP were used to discern the DAL: NEAP (mEq/day) = 54.5 × protein (g/day)/potassium (mEq/day) − 10.2 and PRAL (mEq/day) = 0.49 × protein intake (g/d) + 0.037 × phosphorus (mg/day) − 0.021 × potassium (mg/day) − 0.013 × calcium (mg/day) −0.026 × magnesium (mg/day; Wu et al., 2020).

### 2.6 Statistical analysis

Data analysis was conducted using SPSS version 26 (SPSS Inc., Chicago, IL, USA). The Kolmogorov–Smirnov test was used to determine the normality of the data, and quantitative and qualitative variables were reported as the mean ± standard deviation (*SD*) and number (%), respectively. PRAL and NEAP were divided into quartiles based on the trends. To compare the differences between quantitative and qualitative variables, one‐way analysis of variance (ANOVA), analysis of covariance (ANCOVA), and chi‐square tests were used among quartiles, respectively. Linear regression was used, in crude and adjusted models, to understand the relationship between DAL and pain intensity. Model 1 was adjusted for age, PA, energy intake, BMI, WC, while model 2 was adjusted for model 1 + gender, education, job, marital status, and delivery type. In addition, Spearman correlation was conducted to complement linear regression analyses. Statistical significance was accepted, a priori, at *p* < .05.

## 3 RESULTS

### 3.1 General population characteristics

The mean ± *SD* age, weight, BMI, and pain intensity of participants were 33.23 ± 10.5 (y), 69.35 ± 15.19 (kg), 24.84 ± 4.32 (kg/m²), and 17.06 ± 17.37 across quartiles of DAL, respectively (Table 1). In addition, the mean ± *SD* of NEAP and PRAL were 45.13 ± 24.07 (mEq/day) and −0.43 ± 27.52 (mEq/day), respectively. A significant difference was found between job, marriage, sex, delivery type, age, PA, height, and pain intensity among PRAL quartiles (*p* < .05). Among NEAP quartiles, there was a significant difference in job, marriage, sex, delivery type, age, and height (*p* < .05).

**TABLE 1:** General characteristics of participants among quartiles of NEAP and PRAL

Variables^a^	NEAP (mEq/day)					PRAL (mEq/day)				
	Q1	Q2	Q3	Q4	*p*‐Value	Q1	Q2	Q3	Q4	*p*‐Value
Education (*n*) %										
Diploma or lower	(9) 37.5	(5) 20.8	(5) 20.8	(5) 20.8	.28	(8) 33.3	(4) 16.7	(6) 25	(6) 25	.54
Bachelor's degree	(22) 29.7	(22) 29.7	(14) 18.9	(16) 21.6		(24) 32.4	(19) 25.7	(16) 21.6	(15) 20.3	
Master degree	(9) 20	(8) 17.8	(16) 35.6	(12) 26.7		(7) 15.6	(12) 26.7	(13) 28.9	(13) 28.9	
PhD degree	(4) 12.5	(9) 28.1	(9) 28.1	(10) 31.3		(5) 15.6	(8) 25	(9) 28.1	(10) 31.3	
Job (*n*) %										
Housekeeper	(5) 31.3	(7) 43.8	(2) 12.5	(2) 12.5	.**01**	(4) 25	(6) 37.5	(6)37.5	0	.**05**
Labor	0	(1) 50	(1) 50	0		(1) 50	0	0	(1) 50	
Management employee	(15) 25	(16) 25	(18) 30	(13)20		(15) 24.1	(17) 27.4	(16) 25.8	(14) 22.5	
Nonmanagerial employee	(6) 18.8	(7) 21.9	(6) 18.8	(13) 40.6		(6) 18.8	(5) 15.6	(8) 25	(13) 40.6	
No job	(11) 55	(5) 25	(3) 15	(1) 5		(11) 55	(5) 25	(2) 10	(2) 10	
University student	(7) 16.3	(8) 18.6	(14) 32.6	(14) 32.6		(7) 16.3	(10) 23.3	(12) 27.9	(14) 32.6	
Marriage (*n*) %										
Married	(25) 34.7	(19) 26.4	(17) 23.6	(11) 15.3	.**03**	(28) 38.9	(15) 20.7	(17) 23.6	(12) 16.6	.**02**
Single	(19) 19.4	(25) 25.5	(24) 24.5	(30) 30.6		(16) 16.3	(27) 27.6	(25) 25.5	(30) 30.6	
Divorce	0	0	(3) 60	(2) 40		0	(1) 20	(2) 40	(2) 40	
Gender										
Male	(5) 10	(8) 16	(16) 32		.**001**	(4) 8	(11) 22	(11) 22	(24) 48	**<.001**
Female	(39) 31.2	(36) 28.8	(28) 22.4			(40) 32	(32) 25.6	(33) 26.4	(20) 16	
Delivery type (*n*) %										
Cesarean	(17) 50	(10) 29.4	(3) 8.8	(4) 11.8	.**001**	(17) 50	(9) 26.5	(5) 14.7	(3) 8.8	.**001**
Natural	(4) 33.3	(4) 33.3	(3) 25	(1) 3.3		(4) 33.3	(3) 25	(3) 25	(2) 16.7	
No delivery	(18) 22	(22) 26.8	(24) 29.3	(18) 22		(19) 23.2	(20) 24.4	(26) 31.7	(17) 20.7	
Age y (*n*)%										
18–35	(15) 14.3	(26) 24.8	(29) 27.6	(35) 33.3	.**006**	(15) 14.3	(24) 22.9	(32) 30.5	(34) 32.4	.**001**
36–55	(20) 137.7	(16) 30.2	(13) 24.5	(4) 7.5		(20)37.7	(16) 30.2	(11) 20.8	(6**)** 11.3	
>55	(9) 52.9	(2) 11.8	(2) 11.8	(4) 23.5		(9**)** 52.9	(3) 17.6	(1) 5.9	(4) 23.5	
PA (*n*)%										
High	(21) 27.6	(23) 30.3	(15) 19.7	(17) 22.4	.45	(25) 32.9	(20) 26.3	(12) 15.8	(19) 25	.**01**
Moderate	(3) 15	(3) 15	(7) 35	(7) 35		(3) 15	(2) 10	(5) 25	(10) 50	
Low	(20) 25.3	(18) 22.8	(22) 27.8	(19) 24.1		(16) 20.2	(21) 26.6	(27) 34.2	(15) 19	
Quantities variables^b^										
BMI (kg/m^2)^	24.26 ± 3.97	25.14 ± 4.91	24.55 ± 3.79	25.42 ± 4.59	.57	24.99 ± 4.58	24.76 ± 4.20	24.60 ± 4.15	25.00 ± 4.49	.96
Height (cm)	163.68 ± 7.57	165.33 ± 9.07	167.80 ± 8.15	169.88 ± 9.38	.**005**	163.98 ± 7.63	165.38 ± 8.54	165.75 ± 8.76	171.48 ± 8.65	**<.001**
Weight (kg)	65.19 ± 12.91	69.17 ± 16.79	69.50 ± 14.27	73.65 ± 15.88	.07	67.46 ± 15.04	68.10 ± 14.46	68.16 ± 15.87	73.66 ± 15.06	.18
WC (cm)	83.25 ± 16.05	90.20 ± 24.80	85.55 ± 17.00	87.56 ± 15.27	.35	86.14 ± 18.28	87.93 ± 23.58	86.23 ± 16.97	86.27 ± 15.77	.96
Pain intensity	14.30 ± 16.14	14.36 ± 16.07	19.39 ± 18.76	20.30 ± 18.08	.21	15.82 ± 16.61	13.09 ± 15.44	16.30 ± 18.92	22.98 ± 17.35	.**05**

Abbreviations: BMI, body mass index; NEAP, net endogenous acid production; PA, physical activity, WC, waist circumference; PRAL, potential renal acid load.

^a^ Calculated by chi‐square and analysis of variance (ANOVA) for qualitative and quantitative variables, respectively. Bold values indicates that *P*‐value  < .05 was significant.

^b^ Mean ± *SD*.

### 3.2 Dietary intakes across NEAP and PRAL quartiles

Dietary intakes of participants are shown between quartiles, after adjusting energy intake, in Table 2. The mean± *SD* of total energy intake was 2230 ± 651 (Kcal) and there was a significant difference in potassium, magnesium, vitamin C, B9, and E, and fiber intake among PRAL quartiles (*p* < .05). Energy, protein, carbohydrate, and fat consumption increased across PRAL groups. Furthermore, dietary calcium, potassium, magnesium, vitamin C and B9, carbohydrate, and fiber intake were significantly different among NEAP quartiles (*p* < .05). Protein and sodium intake increased among NEAP quartiles.

**TABLE 2:** Energy‐adjusted dietary intakes and nutrients across two groups of NEAP and PRAL

Variables amounts per day^a^	NEAP (mEq/day)					PRAL (mEq/day)				
	Q1	Q2	Q3	Q4	*p*‐Value	Q1	Q2	Q3	Q4	*p*‐Value
Energy (Kcal)	2184.82 ± 673.49	2096.69 ± 550.39	2254.45 ± 614.65	2391.29 ± 741.81	.19	2274.64 ± 623.00	1993.18 ± 584.60	1998.45 ± 529.56	2651.92 ± 648.98	**<.001**
Minerals										
Calcium (mg/day)	1207.20 ± 622.97	1209.49 ± 567.51	1365.55 ± 478.92	1106.73 ± 537.16	.**004**	1285.23 ± 585.66	1105.67 ± 573.95	1144.41 ± 475.58	1353.65 ± 568.92	.49
Potassium (mg/day)	4593.57 ± 1971.99	3675.58 ± 1195.37	3731.55 ± 1145.43	3174.25 ± 1374.88	**<.001**	4827.73 ± 1817.27	3342.67 ± 1163.59	3157.14 ± 1281.63	3851.25 ± 1244.81	**<.001**
Phosphorus (mg/day)	1462.96 ± 626.30	1478.75 ± 596.40	1679.71 ± 544.72	1629.16 ± 899.23	.37	1533.87 ± 598.68	1326.44 ± 591.94	1403.16 ± 540.45	1980.22 ± 785.15	.24
Magnesium (mg/day)	395.34 ± 179.38	336.57 ± 127.38	339.50 ± 105.01	304.73 ± 135.62	**<.001**	415.77 ± 168.85	306.97 ± 128.14	286.60 ± 109.26	366.85 ± 121.16	**<.001**
Sodium (mg/day)	3590.22 ± 849.46	3758.71 ± 730.85	3965.63 ± 782.04	4327.20 ± 1280.31	.**006**	3723.05 ± 881.85	3702.91 ± 720.67	3732.95 ± 760.68	4468.67 ± 1216.55	.08
Vitamins										
C	183.04 ± 90.91	123.84 ± 50.97	122.83 ± 66.49	85.16 ± 54.12	**<.001**	192.43 ± 83.89	115.86 ± 52.18	100.71 ± 69.27	106.56 ± 54.78	**<.001**
B9	469.44 ± 312.63	346.51 ± 123.71	334.26 ± 125.94	260.28 ± 102.07	**<.001**	486.12 ± 305.13	316.28 ± 123.65	290.61 ± 124.99	318.75 ± 116.74	**<.001**
E	23.68 ± 39.74	22.23 ± 32.69	18.30 ± 26.39	14.74 ± 9.64	.17	20.96 ± 31.59	25.03 ± 42.91	17.88 ± 22.65	15.33 ± 11.05	.**01**
Macronutrients										
Protein (g/day)	74.82 ± 30.33	80.64 ± 26.71	98.46 ± 32.08	134.68 ± 113.08	**<.001**	80.44 ± 28.50	74.14 ± 26.75	82.52 ± 31.31	150.14 ± 104.66	**<.001**
Carbohydrate (g/day)	276.15 ± 121.26	221.75 ± 69.57	354.40 ± 46.17	239.57 ± 124.52	.**002**	232.20 ± 110.28	211.25 ± 69.34	213.90 ± 69.39	263.56 ± 122.42	**<.001**
Fat (g/day)	97.08 ± 45.05	106.24 ± 49.78	105.54 ± 39.30	106.68 ± 27.55	.17	97.91 ± 40.13	102.20 ± 55.16	97.10 ± 33.49	118.24 ± 29.27	.**04**
Fiber (g/day)	23.93 ± 16.66	14.00 ± 4.63	13.05 ± 4.84	9.37 ± 5.08	**<.001**	24.93 ± 16.04	12.40 ± 4.47	11.32 ± 4.87	11.76 ± 5.73	**<.001**

Abbreviations: NEAP, net endogenous acid production; PRAL, potential renal acid load.

Calculated by analysis of variance (ANOVA) and analysis of covariance (ANCOVA).

All the variables, except energy, adjusted for energy intake. Bold values indicates that *P*‐value < .05 was significant.

^a^ Mean ± *SD*.

### 3.3 The relationship between DAL and pain intensity among participants

In Table 3, linear regression showed a positive relationship between PRAL and NEAP and pain intensity in the crude model and adjusted model 1. This remained between both PRAL and NEAP and pain intensity (*β* = 4.67, 95% CI = 2.15–7.19, *p* < .001; *β* = 4.03, 95% CI = 1.49–6.58, *p* = .002), respectively, after adjusting all confounders in model 2. In addition, we saw a negative, weak correlation between age, gender, and PRAL and NEAP. Moreover, MPs pain and PRAL and NEAP had a positive weak correlation (*r* = 0.24, *p* = .001; Table 4).

**TABLE 3:** The association of NEAP and PRAL with pain intensity among subjects

		Pain intensity^a^		
		Β	95% CI	*p*‐Value
NEAP (mEq/day)	Crude	2.30	(0.001–4.61)	.**05**
	*M1*	3.00	(0.55–5.45)	.**01**
	*M2*	4.03	(1.49–6.58)	.**002**
PRAL (mEq/day)	Crude	2.46	(0.17–4.75)	.**03**
	*M1*	3.32	(0.87–5.77)	.**008**
	*M2*	4.67	(2.15–7.19)	**<.001**

Abbreviations: M1: Adjusted for age, PA, energy intake, BMI, body mass index, WC, waist circumference.

M2: Adjusted for model 1+ gender, education, job, marital status, delivery type.

Bold values indicates that *P*‐value  < .05 was significant.

^a^ Linear regression was used; B: the rate of change per unit, CI, confidence interval; PRAL, Potential renal acid load; NEAP, Net endogenous acid production.

**TABLE 4:** The correlation between dietary acid load (DAL) and musculoskeletal pain intensity

Variables	PRAL (mEq/day)		NEAP (mEq/day)	
	*R*	*p*	*R*	*p*
PA (met/h/w)	−0.07	.31	−0.05	.45
Age (y)	−0.33**	**<.001**	−0.32**	**<.001**
Gender	−0.33**	**<.001**	−0.32**	**<.001**
Job status	0.09	.20	0.09	.21
Delivery type	0.12	.11	0.14	.05
Marital status	−0.17*	.**01**	−0.14	.05
Education status	0.15^a^	.**04**	0.18	.**01**
BMI (kg/m^2^)	−0.004	.35	0.06	.37
Energy intake (kcal)	0.18*	.**01**	0.12	.11
MPs intensity	0.24**	.**001**	0.24**	.**001**

Abbreviations: BMI, body mass index; MPs, musculoskeletal pain condition; NEAP, net endogenous acid production; PA, physical activity; PRAL, potential renal acid load.

Analyses were performed based on the Spearman correlation test. Bold values indicates that *P*‐value  < .05 was significant.

* Significant relationship less than .05.

** Significant relationship less than .01.

## 4 DISCUSSION

In this study, for the first time, we assessed the association between pain intensity and both NEAP and PRAL, after adjusting for a comprehensive set of confounders. Accordingly, we noted that for each unit reduction of DAL, a ≃4‐unit reduction in pain intensity was found. Concordant with this study, Totsch et al. found that poor diet quality, which is low in fruits and vegetables (low in potassium, vitamin C, and fiber) and high in processed red meat, may be responsible for reducing nociceptive sensitivity and increasing chronic pain in obese, inflamed, mice (Totsch et al., 2016). The recommended daily intake of fiber is 25 g per day for women and 38 g per day for men (Totsch et al., 2016); however, fiber intake was lower in this study and tended to decrease with higher intakes of DAL.

Some studies have shown a significant relationship between DAL and inflammation. For instance, higher DAL may cause metabolic acidosis, which can lead to the production of various inflammatory markers (Wu et al., 2019). Moreover, increasing pro‐inflammatory cytokines, such as interleukin 1β (IL‐1β), interleukin 6 (IL‐6), and tumor necrosis factor (TNF‐α), and reducing serum level of anti‐inflammatory markers (interleukin 4 (IL‐4), interleukin 10 (IL‐10), interleukin 11 (IL‐11), interleukin 13 (IL‐13), and transforming growth factor‐β (TGF‐β)), are also responsible for nerve injury and feeling pain (Wu et al., 2019). The amount of potassium and magnesium, and vitamin C and B9 decreased constantly across the increasing quartiles of NEAP and PRAL. Potassium is needed for muscle contractions (Elma et al., 2020), while a Mediterranean‐style diet, which is full of potassium, magnesium, and vitamin C and E, can be a protective diet for rheumatoid arthritis (Kaushik et al., 2020). Dietary vitamin C can contribute to antioxidant capacity and improve muscle soreness (Bryer & Goldfarb, 2006). In this study, dietary vitamin C decreased with higher DAL adherence. Interestingly, some previous studies have shown that vitamin B supplementation (such as B9) can alleviate neuropathic pain (Abdelrahman & Hackshaw, 2021). In the present study, a higher consumption of sodium was seen concomitant to a high DAL. It is evident that excess salt intake alters the endothelial function increasing production of TGF‐β and modulating vascular endothelial growth factor C (VEGF‐C) and increasing risk of arthritis. In addition, a greater intake of sodium can increase pain (Salgado et al., 2015). Finally, foods rich in acid‐producing properties are usually low in magnesium; this nutrient can help to eliminate chronic pain due to prolonged opening of calcium channels and activation of N‐methyl‐D‐aspartate (NMDA) receptors, which can remain open in the absence of magnesium (Tarleton et al., 2020).

Aligned with increasing PRAL, protein, carbohydrate, and fat intakes were concurrently elevated. In line with this study, the consumption of high amounts of carbohydrates can reportedly play an important role in oxidative stress, specifically via glucose oxidation (Kaushik et al., 2020). In addition, animal proteins, which are high in methionine, can reduce blood pH and the incidence of musculoskeletal pain (Elma et al., 2020). In contrast with the present study, a prior investigation found a significant positive association between a higher DAL and greater muscle strength. On the other hand consistent with this study, higher PRAL and NEAP scores may be to bone and muscle loss, which may elicit feelings of pain (Chan et al., 2015) through the ubiquitin–proteasome pathway and insulin‐like growth factor‐1 (IGF‐1) signaling (Hayhoe et al., 2020; Mohammadpour et al., 2020). Finally, higher secretion of cortisol and muscle loss may be another probable mechanism of following higher DAL diets (Williamson et al., 2021).

To the best of our knowledge, the current study represents the first to have investigated DAL and MPs intensity. Nevertheless, one of the limitations of the present study is the imbalanced sample, with a disproportionate number of women versus men, which could impact our findings. Women have been posited to report greater pain compared with men due to greater nerve density (Paller et al., 2009). Furthermore, the population of this study was mostly among young adults (18–35 years) and may explain the low mean score of pain intensity. Assessing older adults in the quarantine period of COVID‐19 was logistically impractical, and this may have influenced the final results. In addition, based on the weak associations found, some hidden confounders may have influenced the results. Furthermore, the cross‐sectional design of the study precludes causal inferences being made. Finally, the recall‐based measurements in the present study are dependent on memory, cooperation, and communication ability of the subject, all of which may be subject to bias. Clearly, further studies are needed to ascertain the long‐term impact of DAL on musculoskeletal pain.

## 5 CONCLUSION

This study demonstrates a significant, positive, relationship between DAL and pain intensity among adults with musculoskeletal pain.

## CONFLICTS OF INTEREST

The author declares that there is no competing interest.

## INSTITUTIONAL REVIEW BOARD STATEMENT

The National Committee for Ethics in Biomedical Research approved this study under code IR.IAU.SRB.REC.1399.084. The specifics of the study were told to all qualified participants and written consent was obtained. The data are not publicly available because of containing information that could compromise the privacy of the research. Data are available from the authors upon reasonable request and with permission.

## INFORMED CONSENT STATEMENT

Committee for Ethics in Biomedical Research approved this study under code IR.IAU.SRB.REC.1399.084.

## CONSENT FOR PUBLICATION

The author listed approved the final manuscript and consent for publication.

## Data Availability

Data supporting the results of this study are available from the Islamic Azad University of Science and Research Branch (SRBIAU) and have been used under license for the current analysis. However, data are available from the writers with the permission of the clinics and upon fair requests. It has been stated in our contract between the clinic and us that they never send us details about the participants because our data are part of a great database. Even they have their own competent statistics expert who analyzes our findings, and the results were written based on his report.
